# Magnetization process of a ferromagnetic nanostrip under the influence of a surface acoustic wave

**DOI:** 10.1038/s41598-020-66144-0

**Published:** 2020-06-10

**Authors:** David Castilla, Rocío Yanes, Miguel Sinusía, Gonzalo Fuentes, Javier Grandal, Marco Maicas, Tomás E. G. Álvarez-Arenas, Manuel Muñoz, Luis Torres, Luis López, José L. Prieto

**Affiliations:** 1 Instituto de Sistemas Optoelectrónicos y Microtecnología (ISOM), Universidad Politécnica de Madrid, Avda. Complutense 30, 28040 Madrid, Spain; 2 Dpto. Física Aplicada, University of Salamanca, Plaza de los Caídos S/N, E-37008 Salamanca, Spain; 3 Instituto de Tecnologías Físicas y de la Información (CSIC), Serrano 144, 28006 Madrid, Spain

**Keywords:** Spintronics, Ferromagnetism, Magnetic devices

## Abstract

Surface Acoustic Waves (SAW) are one of the possible solutions to target the challenges faced by modern spintronic devices. The stress carried by the SAW can decrease the current required to achieve magnetic switching or domain wall movement by spin transfer torque. Although the last decade has produced very relevant results in this field, it is still important to study the effects of a SAW on the basic unit of many spintronic devices, a ferromagnetic nanostrip. In this work, we perform a complete set of measurements and simulations to characterize the magnetization process of a Ni nanostrip under the influence of a SAW. We find that the SAW increases the mobility and the depinning ability of the magnetic domain walls and consequently, promotes a sharper approach to saturation and substantially decreases coercivity. We have also found other two interesting effects. When the SAW has sufficient energy, is able to trigger irreversible transitions even before switching the direction of the external magnetic field. Additionally, we have found that the magnetization process depends on the direction of the travelling SAW.

## Introduction

The field of spintronics has recently started to explore the advantages of coupling Surface Acoustic Waves (SAW) with nanomagnetic devices. An interdigitated transducer (IDT) is often the choice to generate SAW’s at the surface of a piezoelectric substrate and, with modern nanolithography techniques, the excitation of SAW in the GHz range is quite standard. These frequencies are within the range of spin-wave resonance or spin precession, which opens the door to complex and potentially very useful interactions between the stress carried by the SAW and the magnetoelastic nature of the ferromagnetic material. From fundamental point of view, SAWs have been used to promote elastically driven ferromagnetic resonance in a thin film^1,2^, to pump spin current^3^, to detect delayed magneto-dynamic modes^4^ or magnon-phonon dynamics^5^, or even to measure the intrinsic Gilbert damping of a single nanomagnet^6^. From a more practical point of view, SAW’s have been shown to promote the switching in ferromagnetic films^7^ or nano-elements^8,9^, to promote magnetization rotations in ferromagnetic bars^10,11^, to assist magnetic recording^12^ or to reduce the energy dissipation in spin-transfer-torque random access memories^13^. Additionally, as many of the modern spintronic devices are based on magnetic domain walls (DW), there have been interesting proposals to use SAWs to control the movement of DWs^14^ and indeed there has been a recent experimental demonstration of SAW assisted DW motion in films with perpendicular magnetic anisotropy^15^. Nevertheless, despite being the ferromagnetic nanostrips the fundamental unit of many spintronic nano-devices, to our knowledge, there are still no experimental studies dealing with the interactions between SAWs and DWs in nanostrips. In this work, we explore the effect of SAWs on the magnetization process of a Nickel ferromagnetic nanostrip. Our experimental results show how the SAW assists the movement and depinning of the DWs during the magnetization process in the nanostrip. This translates into an important reduction of the coercivity in the nanostrip and a sharper approach to saturation. Additionally, we show that the SAW can promote the nucleation and movement of DWs that initiate the magnetic reversal, before switching the direction of the external magnetic field. Finally, and importantly, we found that the movement of the DWs under the action of the SAW is not symmetric with the direction of the traveling SAW. We have fitted the experimental results with micromagnetic simulations to gain some inside into how the stress carried by the SAW interacts with the DW.

## Results and Discussion

### Experimental set-up and measurements

**Figure 1:**
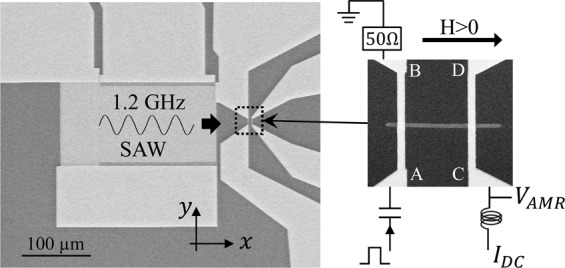
SEM photograph on the left showing the Interdigital Transducer (IDT) that produces the Surface Acoustic Wave. The right picture shows a zoom-in of the nanostrip with the connections for the measurements described in this work.

The device used in this work couples a SAW emitter, deposited over a ScAlN piezoelectric film, with an adjacent Nickel ferromagnetic nanostrip, as shown in Fig. 1. The Ni ferromagnetic nanostrip, 400 nm wide and 10 µm long, with the structure Cr(4 nm)/Ni(35 nm)/Pt(2 nm), is 40 µm away from the edge of the IDT and with its long axis aligned with the direction of the travelling SAW. The ferromagnetic nanostrip has four electric contacts, visible in Fig. 1. The DC current used to measure the Anisotropic Magnetoresistance (AMR) of the stripe flows from contact C to ground and its value is 100 µA. This current is too small to generate any heat or any spin transfer torque in the Ni nanostrip. Additionally, in some experiments, we have used current line A-B to generate a pulse of local magnetic field by injecting a current pulse from contact A to B. For more details, refer to Methods.

**Figure 2:**
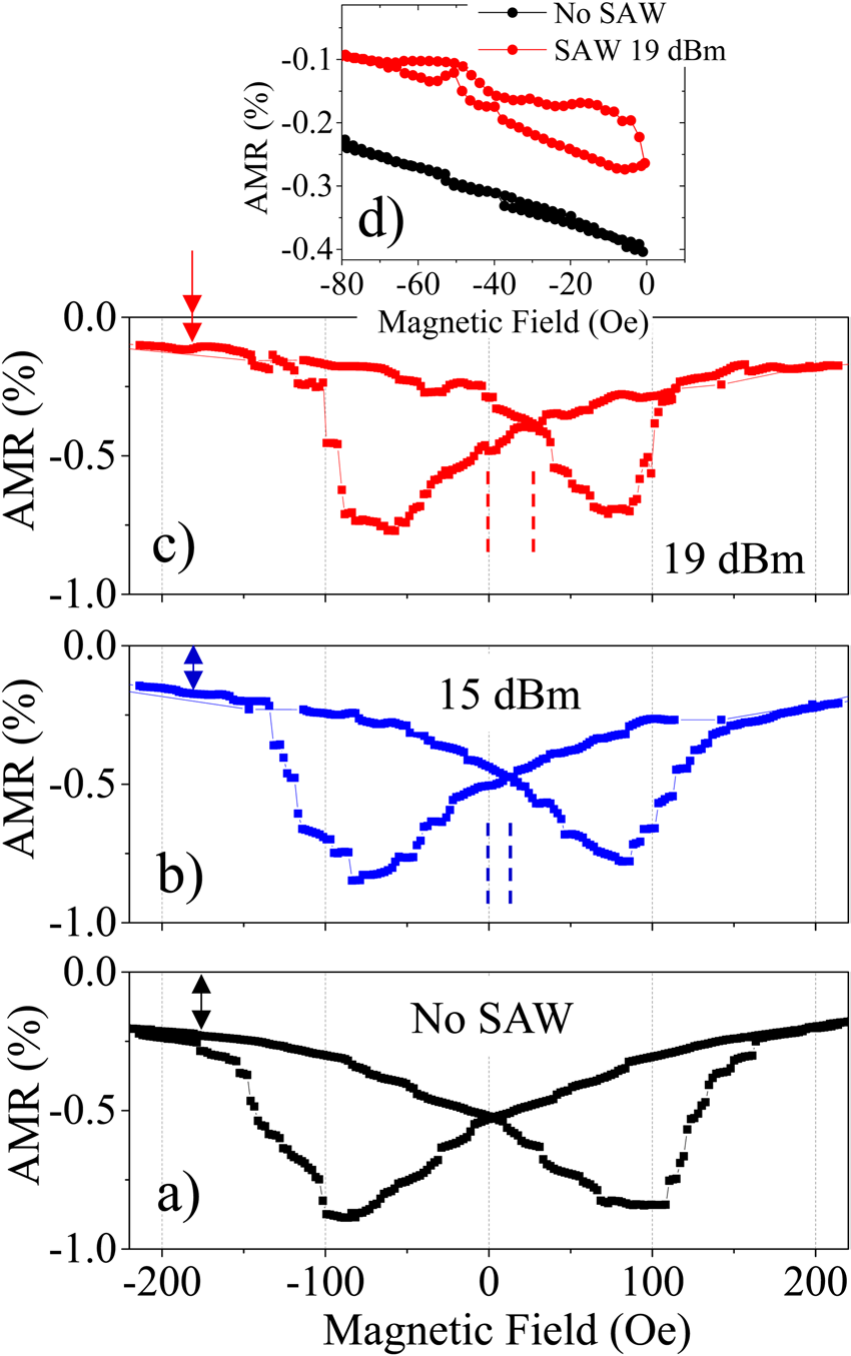
(**a**–**c**) Show the central area of an anistotropic magnetoresistance (AMR) loop without SAW (black) and for 15 dBm (blue) and 19 dBm (red) SAW power. In (**d**), we present two minor loops with and without SAW, showing that the SAW can trigger an irreversible behaviour even before switching the direction of the external magnetic field.

Figure 2 shows the Anisotropic Magnetoresistance (AMR) loops of the Ni nanostrip, with the field applied along the nanostrip long axis, for different amplitudes of the SAW. The maximum applied field reaches ± 1000 Oe, but we only show a zoom of the central part of the loop to highlight the relevant features. All the cycles reach 0% AMR at saturation (±1000 Oe), when the magnetization is perfectly aligned with the nanostrip axis and the direction of the current flow. It is important to note that our Ni nanostrips show an effective uniaxial anisotropy direction at 55° with respect to long axis to the nanostrip, as shown in Supplementary Information Figs. S1 and section S4. This angle allows an almost optimal interaction between the ferromagnetic material and the SAW^1^.

Figure 2 reveals several interesting consequences of coupling the SAW to the magnetization process of the Ni nanostrip. Firstly, it is obvious that the SAW promotes a reduction of the coercivity (Fig. 2a–c). This reduction is a consequence of the SAW providing an alternative source of energy (magnetoelastic) for the DWs to overcome local pinning barriers. Back in the 1990’s, the hysteresis of magnetic sensors was reduced using piezoelectric actuation^16^. More recently, we can find reports on SAW induced coercivity reduction of up to 11% in a highly magnetostrictive Galfenol film^12^ or even up to 60% in an epitaxial ferromagnetic semiconductor^17^ (measured at a low temperature of 38 K). In this context, the room temperature coercivity reduction of 17% visible in Fig. 2 is remarkable, specially taking into account the moderate saturation magnetostriction of Nickel. As we will see with micromagnetic simulations, the origin of this coercivity reduction is the large magnetoelastic energy delivered by this piezoelectric, that ultimately translates into a very effective DW depinning induced by the SAW.

The second interesting feature visible in Fig. 2 is that, as the energy of the SAW increases, the approach to saturation is sharper. The double arrow on the top left corner in Fig. 2a–c indicates how the AMR value gets closer to saturation (0% AMR) as the SAW power increases. Additionally, in the branch that goes from the coercitive field to saturation, the transition is much more abrupt when the power of the SAW is larger (compare for instance the transition around −100 Oe between the black and the red curves in Fig. 2). There is also an obvious asymmetry in the AMR loop induced by the SAW, indicated by a double dashed line around zero magnetic field in the blue and red loops. The origin of this asymmetry is not completely understood but it is not present when measuring the AMR loop with an AC current (Supplementary Information Section S2). Therefore, it may be related to the acoustoelectric effect^18^, a unidirectional build-up of charges in the nanostrip dragged by the acoustic wave.

Finally, Fig. 2d displays another important feature triggered by the SAW. In this figure, we show two minor loops, without SAW (black) and with 19 dBm SAW (red). The SAW leads to a non-reversible minor loop even if the field does not switch direction. This can be achieved only for a power larger than 15 dBm and it happens in all the nanostrips measured, even in nanostrips deposited in different conditions with slightly smaller average grain size (11 nm) as it can be seen in in Supplementary Information Section S3. This irreversible minor loop indicates that the SAW can assist the nucleation and/or the movement of magnetic domain walls, even before switching the direction of the external field.

### Micromagnetic simulations and magnetization process

In order to gain some insight into the effect of SAW on the magnetic response of our Ni sample, we have performed MuMax^19^ micromagnetic simulations on a ferromagnetic nanostrip of the same dimensions as the experimental device (10 µm × 400 nm × 35 nm). The strip was divided in 2048 × 64 × 1 cells with a cell size of 4.95 × 6.25 × 35 nm^3^. We obtained the same results when performing the simulations using 2, 3 and 4 cells along the out-of-plane direction. The polycrystalline grain structure was modelled via a Voronoi tessellation of the system in polygonal regions of average diameter *d* = 20 nm. A nominal value *M*_*s*_ = 2.38·10^5^ A·m^−1^ with a 4% dispersion among grains is chosen for the saturation magnetization. The exchange constant is *A*=1.05·10^−11^ J·m^−1^ and a 20% reduction at the grain boundaries is assumed. In addition, we tried several anisotropy configurations in the nanostrip with angles 0°, 30°, 60° and 90° with the nanostrip axis. In order to reproduce accurately the experimental AMR loop, we had to assume a small uniaxial anisotropy of *K* = 6.0·10^3^ J·m^−3^ in the *y* direction (90°), with a dispersion of 11.4° (see also Supplementary Information S4). This anisotropy, together with the shape anisotropy in the *x* direction, results in an effective uniaxial anisotropy at ~60° with the nanostrip axis, not far from the 55° measured experimentally. For the calculation of the AMR signal from the results of the simulation, we have assumed that the current flows perfectly along the nanostrip. The AMR signal is obtained from the average of the squared longitudinal component of the magnetization $< {m}_{x}^{2} >$, namely ${\rm{A}}{\rm{M}}{\rm{R}}({\rm{ \% }})=(\langle {m}_{x}{\rangle }^{2}-1)({R}_{||}-{R}_{\perp }) / {R}_{||},$ where ${R}_{||}({R}_{\perp })$ is the resistance of the device when the magnetization is perfectly parallel (perpendicular) to the current density.

**Figure 3:**
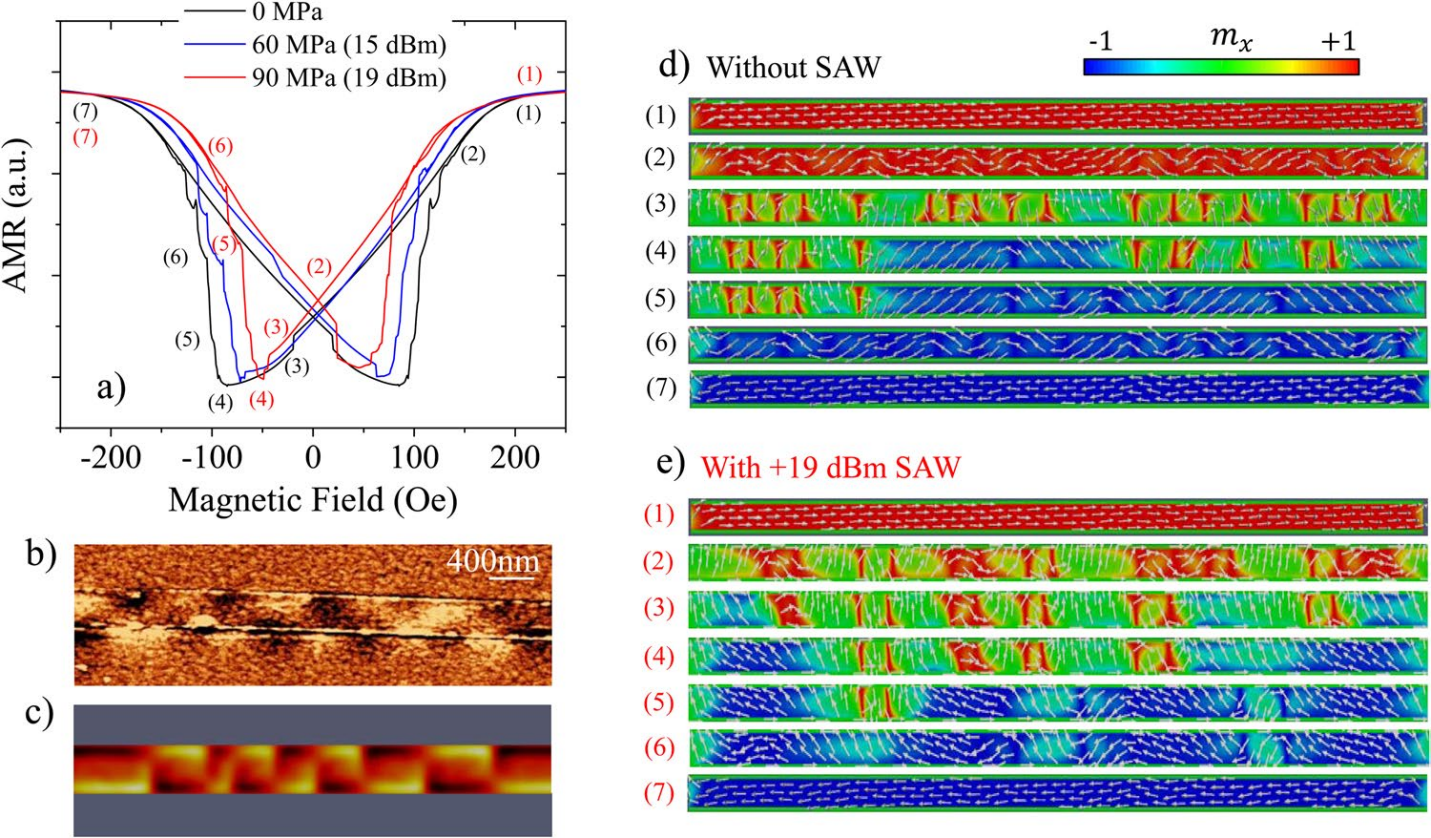
(**a**) Shows the AMR loops obtained through micromagnetic simulations for the SAW powers used in the experiment. (**b**) MFM image of the Ni nanostrip at remanence with no SAW. (**c**) Simulated MFM signal at remanence with no SAW. (**d**) Snapshots of the magnetization process at different fields without SAW and (**e**) with SAW. The numbers indicate the position in the AMR loop in (**a**) at which the snapshot is taken. The colour scale on the top indicates the direction of the *x*-component of the magnetization.

The black curve in Fig. 3 shows the AMR loop computed numerically without any SAW excited. The simulated AMR loop is in good agreement with the experimental one (Fig. 2a), displaying a gradual resistance decrease and a few small jumps before the minimum at the coercive field (${H}_{c}\simeq 100\,{\rm{Oe}}$), followed by a more pronounced increase with several larger irreversible jumps up to saturation. Before discussing the complex reversal mechanism described by the simulations, we have compared the experimental domain configuration at remanence, measured with magnetic force microscopy (Fig. 3b), with the configuration predicted by the simulations (Fig. 3c). The qualitative resemblance of the simulated and experimental configurations is very good, showing a multidomain configuration. A very similar multidomain configuration has also been observed in microstrips of a different magnetostrictive material^20^. Figure 3d,e show snapshots at different fields during the magnetization process of the nanostrip, both with and without SAW (see also the animation in Supplementary Material). When there is no SAW (Fig. 3d.), as the field decreases from positive saturation, the system breaks into multiple domains that tend to orientate along the *y*-axis, either positive or negative, separated by domain walls pointing along the positive *x* direction. When the field becomes negative, two reversal mechanisms occur: gradual rotation in the domains and domain wall displacement. The jumps in the AMR loop correspond to irreversible events where two or more domain walls collapse with each other.

In order to implement the effect of the SAW in the simulation of the magnetization dynamics, we added a magneto-elastic contribution to the effective field in the Landau-Lifshitz-Gilbert equation, as described in Methods. In Fig. 3, we compare the AMR loop without SAW (black) together with those obtained with a SAW power 15 dBm and 19 dBm (see conversion from dBm to MPa in Supplementary Information Section S6). The external field was ramped in steps of 1 mT, maintaining each field for a time interval ${t}_{w}=100\,{\rm{ns}}$ and with α = 0.01 as damping constant. This simulation does not account for thermal fluctuations, but including them did not produce any significant changes.

Figure 3 shows that our simulations reproduce most of the features observed experimentally (Fig. 2a–c). The SAW promotes a clear reduction of the coercivity, even larger than in the experiment. This was expected because in the simulation, all the energy carried by the SAW is delivered to the nanostripe, which is not the case in the experiment. In addition, the approach to saturation is more abrupt, with fewer but more pronounced jumps, as it also happens in the experiment. Finally, the simulations also reproduce that the slope of the initial reversible part of the loop, just below saturation, is smaller with SAW. All these features can be understood considering that, in a first approximation, the effect of SAW is equivalent to a field along the *x* direction whose strength is proportional to *m*_*x*_ (${H}_{me}^{x}\approx {\lambda }_{s}{\sigma }_{x}{m}_{x}/{\mu }_{0}{M}_{S}$), thus favouring longitudinal orientation of the spins. As the field decreases from saturation and the system breaks into domains oriented along the *y* direction, the DWs separating them become wider when the SAW is applied. When two of these wider DWs are close to each other, they tend to merge into a single extended region predominantly oriented along the positive *x* direction (see snapshots in Fig. 3 and animation in Supplementary Material). Consequently, fewer *y*-oriented domains are present in the system with wider *x*-oriented DWs separating them, which leads to a smaller slope in the initial reversible part in the loop and a higher remanence. Also, since there are less domains, irreversible jumps are scarce but more pronounced. In particular, around the coercive field there are a few large irreversible jumps, corresponding to domain wall displacements over long distances and subsequent annihilation (see animation in Supplementary Material). The SAW also introduces a periodic excitation in the domain walls that favours depinning. This is the microscopic mechanism behind the reduction in the coercivity. Finally, once all the irreversible jumps have taken place, the SAW also facilitates the alignment of the magnetization with the longitudinal field, which gives a more abrupt approach to saturation.

**Figure 4:**
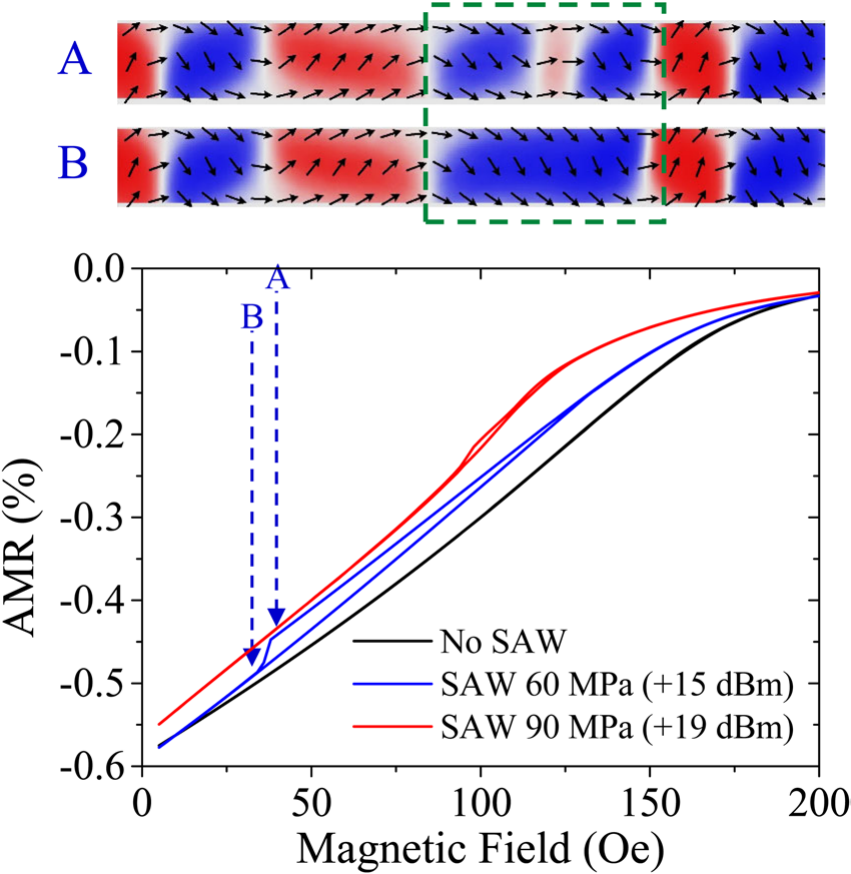
The bottom plot shows and example where the simulation could reproduce the experimental hysteretic minor loop of Fig. 2d. The minor loops run from positive saturation to a positive small field (the direction of the external magnetic field does not change throughout the minor loop), and they only show hysteretic behaviour (red and blue curves) when sufficiently energetic SAW is excited by the adjacent IDT. When there is no SAW flowing in the device, the minor loop is completely reversible (black curve). The snapshots on top of the figure show the magnetic configuration of a section of the nanostrip before and after the hysteretic jump. In these two snapshots there is an area highlighted by a green dashed square where two magnetic domain walls, have been pushed by the surface acoustic wave to collapse with each other.

We now turn our attention to the irreversible minor loop displayed in Fig. 2d. In that figure we saw how the stress carried by the surface acoustic wave is able to trigger an irreversible magnetic transition even before the external magnetic field switches direction. This irreversible minor loop has no easy explanation in the light of results reported by other authors. Although mechanical stress can rotate the magnetic easy axis^21^ by 90° or achieve irreversible 90° rotation by applying static stress at 45° with two orthogonal magnetic easy axis^22^, our simulations show that these effects cannot justify an irreversible magnetic transition before switching the direction of the external magnetic field. As shown in Fig. 4, our simulations manage to reproduce an irreversible jump in a minor loop similar to the experimental one in Fig. 2d. The irreversible jump may (or may not) occur for each field value depending on the time we allow for the magnetization to relax to its equilibrium configuration. In the particular case displayed in Fig. 4, we managed an irreversible minor loop using field steps of 2 Oe, 4 ns relaxation time and a damping constant α = 1. The snapshots of a section of the nanostrip (top of Fig. 4) show how, in these particular conditions, the SAW is able to make two DWs collapse and disappear (marked with a dashed square), generating the jump in the AMR value. Therefore, the experimental finding shown in Fig. 2d, seems to be a consequence of the SAW affecting metastable magnetic configurations achieved in the nanostrip as the field is ramped. Obtaining the exact conditions that allow a ferromagnetic nanostructure, under the action of a SAW, to make large irreversible transitions such as the one shown in Supplementary Information Fig. S3.2, is beyond the scope of this work.

### The role of the SAW in the depinning of the magnetic domain wall

As explained in the introduction, one of the goals behind using SAW in modern spintronic devices is to assist the movement and depinning of DWs. Although in the previous section we studied the magnetization process both with and without SAW, we still need to explore further into the movement and depinning of DWs under the action of the SAW and determine to what degree our micromagnetic model captures all the experimental particularities.

**Figure 5:**
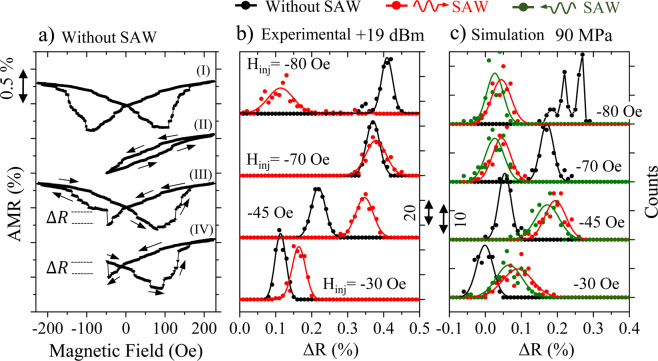
(**a**) shows the AMR loop without any SAW labelled as (I), which is the same as in Fig. 2a, and several minor loops (without any SAW) as described in the main text, labelled as (II), (III) and (IV). In (**b**) we show histograms of the jump in the AMR, labelled as ΔR in the blue loop in (**a**), without SAW (black) and with SAW (red) for different injection field in the negative *x-*direction. (**c**) shows the corresponding simulated histograms, without SAW (black), with SAW flowing towards the right, like in the experiment (red). We have added a simulation with the SAW flowing towards the left (green), in the opposite direction to the one used in the experiment. The box on top of the figure shows the colour code for the different experiments and simulations.

In Fig. 5a we show a sequence of AMR loops and minor loops (all without SAW). The top curve, labelled as (I) is in fact the same one shown in Fig. 2a, where we will perform minor loops starting from positive saturation (+1000 Oe), as indicated by the arrows. Any minor loop performed in the Ni nanostrip without SAW, would be reversible until reversal fields larger than −30 Oe (curve labelled as (II) in Fig. 5a). This is an indication that, for reversal fields larger than −30 Oe, there are irreversible movements of magnetic domain walls or irreversible rearrangements of the magnetic domains.

In order to study the movement of the DWs we have performed the following experiment. At any given point of the reversal sequence, we assist the magnetization process with a pulse of local field. For instance, in the curve labelled as (III) in Fig. 5a, we ramp the field from positive saturation to −45 Oe and, at that field, we inject a 15 ns pulse of current (6·10^7^ A/cm^2^) from contact A to contact B. The local Oersted field generated by a 6·10^7^ A/cm^2^ current pulse flowing from contact A to B, is of the order of 400 Oe just underneath the current line^23^. This field provides a controlled push to the DWs and triggers a sudden jump of the AMR in the nanostrip. The amplitude of this jump gives an idea of how large is the distance freed by the DW between two pinning sites.

In order to make some statistics in this experiment, we have repeated the same sequence 200 times: positive saturation, reduce the field slowly to a particular negative field (which we denote as H_inj_), deliver a magnetic field pulse, and record the irreversible AMR jump. In Fig. 5b we can see the histograms with the 200 events for different H_inj_, with no SAW (black curves) and with 19 dBm SAW (red curves). For H_inj_ = −30 Oe the AMR jumps induced by the current pulse are quite small and slightly larger when the SAW is acting. This makes sense by looking at the loops in Fig. 2. For H_inj_ = −30 Oe, the reversal process is still very gradual and a sudden pulse of field should not change the AMR much. For H_inj_ = −45 Oe, the AMR jump induced by the pulse is about double than the one at H_inj_ = −30 Oe in both cases, with and without SAW. For H_inj_ = −70 Oe the AMR jump is equivalent weather there is SAW or not. Finally, for H_inj_ = −80 Oe, the situation reverses: when the SAW is acting the AMR jump is very small, while it is quite notorious when there is no SAW. By looking to the AMR loops in Fig. 2, we can see that when we apply a 19 dBm SAW (Fig. 2c) and for H_inj_ = −80 Oe, the reversal process is about to go through two very large Barkhausen jumps. It seems like the Oersted field associated to the current pulse is not able to deliver enough energy to move the DW across those two big jumps.

We also performed systematic micromagnetic simulations to reproduce the data measured in Fig. 5b, following the same routine as in the experiment (see more details in Methods). Figure 5c shows the main results of these simulations. For each field H_inj_ we computed 100 realizations both without SAW (black data) and with 90 MPa (~19 dBm) SAW travelling in the same direction as in the experiment (red data) and in the opposite direction to the one used the experiment (green data). We considered the same values of H_inj_ than in the experiment. Figure 5c shows that the simulation reproduces several experimental features. First, the perturbation introduced by the SAW favours the occurrence of irreversible processes and, consequently, it broadens the statistical distribution of ΔR. Secondly, in general, the average ΔR is larger with SAW, indicating that, as already mentioned, SAW induce larger displacements of the DWs. Thirdly, for higher fields, there is a reversal of the tendency and the ΔR jump is smaller with SAW than without SAW. This is understandable since, for those fields, the reversal of the nanostrip is close to completion (80 Oe in the experiment and 70 Oe in the simulation for 19 dBm). Therefore, for large fields, a pulse of Oersted field does not contribute to the magnetization process in the almost saturated nanostrip when the SAW is acting but it does when there is no SAW, as the strip is still far from saturation.

Finally, it is important to pay close attention to the green histograms in Fig. 5c. Here we repeated the statistical study with the SAW propagating in the opposite direction to the one stablished in the experiment, i.e. towards the left. As can be observed, we obtain a very similar statistical distribution, although slightly displaced towards smaller ΔR in all cases. It is a small effect but unmistakable in the micromagnetic simulations. The DW displacement depends on the direction of travel (+*x* or −*x* direction) of the SAW. This mechanism demands experimental confirmation and further investigation to understand its origin, which could be due to transfer of linear momentum between elastic and magnetic subsystems. With proper design of the device, this effect may become sizable and it could add functionality to modern spintronic devices by making the magnetization process largely dependent on the direction of travelling of the SAW.

## Conclusions

We have completed a detailed study of the magnetization process of a Ni nanostrip under the action of a surface acoustic wave. The experiment has shown how the SAW induces an effective reduction of the coercivity and a sharper approach to saturation. Additionally, high power SAW are able to induce hysteretic transitions in the magnetization process even before switching the direction of the external magnetic field. Our micromagnetic simulations were able to reproduce all these features, allowing us to picture the internal mechanisms behind the SAW-assisted magnetization process. The stress carried by the SAW provides magnetoelastic energy that helps the movement and depinning of the DWs. This translates at the macroscopic level as a reduction of the coercivity. Additionally, this study helped stablishing two interesting findings. On one hand, highly energetic SAW can induce irreversible transitions even if the external field does no change direction. The simulations show that the SAW is able to affect metastable configurations while the external field is ramped down from saturation. Finally, the simulations have shown that the magnetization process is not symmetric on the direction of the travelling SAW. The SAW is more effective moving the DWs when they travel in the same direction as the SAW.

## Methods

### Fabrication of the nickel nanostrips

The fabrication of the device started with the sputtering deposition of a 2 µm ScAlN piezoelectric film on a Si/SiO_2_ substrate. Before any further processing, the crystalline quality of the ScAlN film was tested by X-Ray Diffraction (θ/2θ and rocking curves) and the piezoelectric response was also measured, being d_33_ = −26 pC/N. After the characterization of the piezoelectric film, we deposited a ferromagnetic 10 µm × 400 nm Nickel nanostrip on top, patterned with e-beam lithography and a standard lift-off process. The structure of the nanostrip was Cr(4)/Ni(35)/Pt(2), with the numbers indicating the thickness in nanometers. The electric resistivity of the Ni is 18.6·10^−8^ Ω·m. We tested the crystalline structure of the Nickel by X-Ray Diffraction (XRD) on a thin film deposited in parallel with the Ni nanostrip. The nickel film grows polycrystalline with a grain size smaller than 20 nm textured in the <111> direction. We can alter this grain size with the deposition conditions and some examples in Supplementary Information refer to nanostrips with different grain size. We indicate the different grain size when this is the case.

Finally, the interdigital transducer to generate the SAW and the electric contacts of the Ni nanostrip, where deposited on a single lithography step, with the structure Cr(20 nm)/Au(120 nm). With a Network Analyser, we found two resonance peaks in the SAW device, the Rayleigh and Sezawa modes. The Rayleigh mode, at 1.2 GHz, is the one used in all the experiments described in this work. The measured reflected power is in the range of −20 dBm to −30 dBm, therefore most of the power delivered by the signal generator is transmitted to the device.

### Integrating the SAW in the simulations

In order to implement the effect of the SAW in the simulation of the magnetization dynamics, we added a magneto-elastic contribution to the effective field in the Landau-Lifshitz-Gilbert equation, [see Supplementary Information Section S5]

$${\overrightarrow{H}}_{me}=\frac{1}{{\mu }_{0}{M}_{s}}\hat{\sigma }\cdot \frac{\partial {\hat{\varepsilon }}^{m}}{\partial \overrightarrow{m}}$$

where $\hat{\sigma }$ and ${\hat{\varepsilon }}^{m}$ are the stress and magnetic strain tensors, respectively. ${\hat{\varepsilon }}^{m}$ is quadratic on the magnetization via the magneto-mechanical coupling tensor. For polycrystalline Ni the magneto-elastic field simplifies to

${\overrightarrow{H}}_{me}=\frac{3}{2}\frac{{\lambda }_{S}\cdot \hat{\sigma }}{{\mu }_{0}{M}_{s}}\overrightarrow{m}$where we have used an isotropic magnetostriction of ${\lambda }_{S}=-\,3.28\cdot {10}^{-5}$, where this value has been calculated as ${\lambda }_{S}=(2{\lambda }_{100}+3{\lambda }_{111})/5$. Micromagnetic simulations were performed using the software package MuMax, modified to include the magneto-elastic contribution ${\overrightarrow{H}}_{me}$ from a spatially non-uniform and time dependent stress. The Rayleigh SAW propagating in the device is modelled via a space and time dependent stress given by

$$(\begin{array}{c}{\sigma }_{xx}\\ {\sigma }_{yy}\\ {\sigma }_{zz}\\ {\sigma }_{zy}\\ {\sigma }_{xz}\\ {\sigma }_{xy}\end{array})={\sigma }_{o}(\begin{array}{c}\sin (kx-\omega t)\\ 0\\ -\,\sin (kx-\omega t)\\ 0\\ 3/8\,\sin (kx-\omega t+\pi /2)\\ 0\end{array})$$

where ${\sigma }_{o}$, *ω* and *k* are the amplitude, frequency and wave number of the SAW, respectively. The values $\omega /2\pi =1.2\,{\rm{GHz}}$ and $k=2\pi /\lambda =2.24\,{{\rm{\mu }}{\rm{m}}}^{-1}$. Frequency values from 0.2 to 2.5 GHz, although not available in the experiment, were tested in the simulations finding qualitatively similar results. Other values can be found in Supplementary Information Section S6.

### Details of the micromagnetic simulations performed to reproduce the experimental histograms

For this simulation, we followed the same routine as in the experiment. Starting from positive saturation, the field is decreased up to a negative value H_inj_ for which some domain walls are already present Fig. 5b. Once equilibrium is reached at that field, a 15 ns current pulse of 6·10^7^ A/cm^2^ is delivered between contacts A and B and the evolution of the system is investigated after the pulse is switched off for 15 ns. The Oersted field created by the current pulse is included as an additional term in the effective field.

As mentioned before, the jump in the AMR (ΔR) is due to the irreversible displacement of domain walls, pushed by the Oersted field. This Oersted field acts locally, mostly in the region underneath the current line. The amplitude of ΔR, consequently, will strongly depend on the magnetization configuration in this region, which itself depends on the sample microstructure (grain distribution, etc.). Since we do not have any knowledge of the experimental local grain distribution, we carried out a statistical study where we investigate the effect of the current pulse over many possible configurations of the system. To do that, we generate different realizations by moving the position of the current line along the length of the nanostrip. Since this length is much larger than both the typical grain size and the width of the current line, this procedure has a similar effect than considering a different grain distribution for each realization. To generate the 100 realizations, the current line is displaced in steps of 60 nm along the central part of the nanostrip, always keeping a minimum distance of 2 µm from the edge. The main advantage of our approach is that the hysteresis curve from saturation to H_inj_, before applying the current pulse, needs to be computed only once, whereas in the other case it would have to be computed for each distribution.

## Supplementary information

Supplementary information.

Supplementary information 2.
