# Supplementary material for: Magnetization process of a ferromagnetic nanostrip under the influence of a surface acoustic wave
Source: Sci Rep. 2020 Jun 10;10:9413. doi: 10.1038/s41598-020-66144-0 (PMC7287114; doi:10.1038/s41598-020-66144-0)
Supplement: Supplementary file 1 — Supplementary information. [file 41598_2020_66144_MOESM1_ESM.pdf]

Magnetization process of a ferromagnetic nanostrip under the influence of a surface acoustic wave

David Castilla[1], Rocío Yanes[2], Miguel Sinusía[1], Gonzalo Fuentes[1], Javier Grandal[1], Marco Maicas[1], Tomás E.G. Álvarez-Arenas[3], Manuel Muñoz[4], Luis Torres[2], Luis López[2], José L. Prieto[1].

[1] Instituto de Sistemas Optoelectrónicos y Microtecnología (ISOM), Universidad Politécnica de Madrid, Avda. Complutense 30, 28040 Madrid, Spain.

[2] Dpto. Física Aplicada. University of Salamanca, Plaza de los Caídos S/N, E-37008, Salamanca, Spain.

[3] Instituto de Tecnologías Físicas y de la Información (CSIC). Serrano 144. 28006, Madrid, Spain.

[4]IMN-Instituto de Micro y Nanotecnología, (CNM-CSIC), Isaac Newton 8, 28760 Tres Cantos, Madrid, Spain

S1. Direction of anisotropy in the Nickel nanostrips

The deposition of the Ni nanostrip in the sputtering was done under a strong magnetic field pointing along the nanostrip long axis. Despite this, the main anisotropy axis in the nanostrip is always at a large angle with respect to the nanostrip axis. In Figure S1 we show AMR loops for different directions with respect to the strip axis. The anisotropy axis is at approximately 55º with respect to the stripe axis.

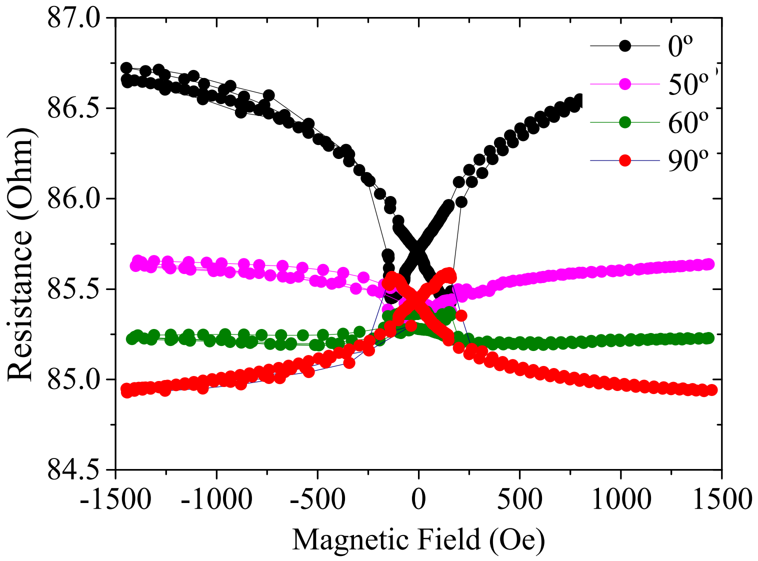

Figure S1. Anisotropic Magnetoresistance curves measured with the external field applied at different angles with respect to the nanostrip axis. The effective uniaxial anisotropy axis is at approximately at 55º with respect to the strip axis.

S2. AMR curves measured with AC current.

We have measured the AMR curves in the same sample used for the loops displayed in Fig. 2 of the main text, but using an AC current and a Lock-in technique for the measurement. Figure S2.1 shows the AMR curve with and without SAW. The Barkhausen jumps are now less obvious. This is due to the fact that the Lock-in integration was done over 1s, which is about the same time that it takes for the external magnetic field to step up to the next value. Therefore, effectively we are averaging out some of the metastable Barkhausen resistance jumps.

It is also noticeable that we have lost the asymmetry of the loop around zero field (highlighted with dashed lines around H=0 Oe in Fig. 2 in the main text). This reinforces the idea that the asymmetry of the AMR loop measured with DC may be related to the acoustoelectric effect.

Finally, as the AC Lock-in resistance measurement cancels out the DC voltage build up coming from the acoustoelectric effect, we are able to estimate the temperature increase due to the SAW by the change of the electrical resistance in the Ni nanostrip. The resistance at saturation with no SAW is 92.2068 Ω and with +19 dBm SAW is 98.2465 Ω. Therefore, if we take $\rho(T) = \rho_0[1+\alpha(T-T_0)]$ with T_0=300 K and the temperature coefficient for Ni, α=0.006 K^{-1}, we can quickly calculate

$$\begin{cases} 98.2465 = \rho(T)\cdot\frac{\ell}{A} = \rho_0[1+\alpha(T-T_0)]\cdot\frac{\ell}{A} \\ 92.2068 = \rho_0\cdot\frac{\ell}{A} \end{cases}$$

Dividing both equations,

$$\frac{98.2465}{92.2068} = 1 + \alpha\cdot\Delta T$$

Therefore $\Delta T = 10.9$ K

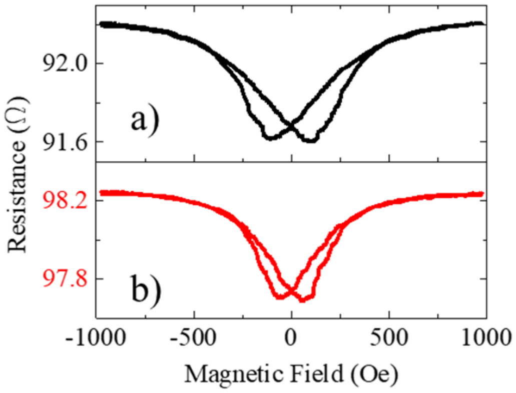

Figure S2.1. AMR curves of the sample used for Fig. 2 in the main text but measured with an AC current and a Lock-in technique. (a) without SAW and (b) with 19 dBm SAW

S3. Irreversible minor loop.

We also studied the effect of SAW in Ni nanostrips deposited in different conditions so the average grain size was 11 nm, slightly smaller than the grain size in the samples used in the main text. In Figure S3.1 we see the AMR plots in this nanostrip, showing very large Barckhousen jumps. The samples, like the ones in the main text, show a uniaxial anisotropy around 55º with respect to the nanostrip axis. The reduction of the coercive field with SAW is also quite notorious.

Note also that the SAW induces large Barkhausen jumps even before the external field switches its direction.

In Figure S3.2 we show a minor loop in these samples with average grain size is 11 nm. The minor loop is taken from positive saturation to +6 Oe (the external field does not switch its direction). The black curve is the minor loop without SAW, showing an almost perfect reversible behaviour. The red curve is the same minor loop but applying +15 dBm SAW in the configuration shown in the main text. The hysteretic behaviour is quite marked when the SAW is applied. With +19 dBm SAW, the hysteretic behaviour persists. This reinforces the idea introduced in the text that, under the action of large amplitude SAWs, there is a mechanism that allows the nucleation of DWs before switching the direction of the external magnetic field.

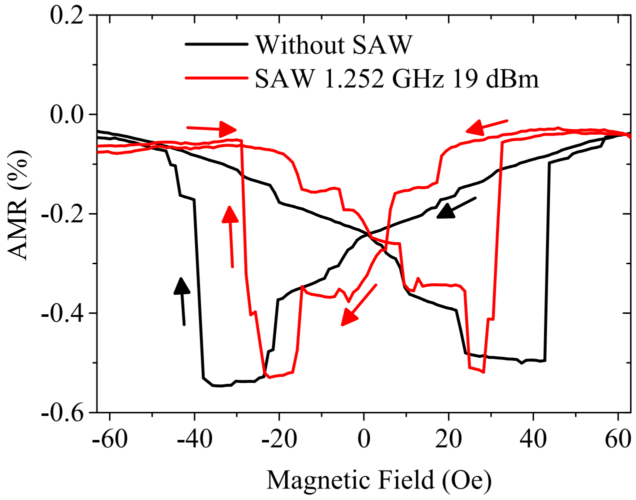

Figure S3.1. Anisotropic Magnetoresistance curves measured with the external field applied along the nanostrip axis without (black) and with +19 dBm SAW (red). The arrows indicate the direction of the magnetization cycle. The reduction of the coercivity with SAW is quite substantial.

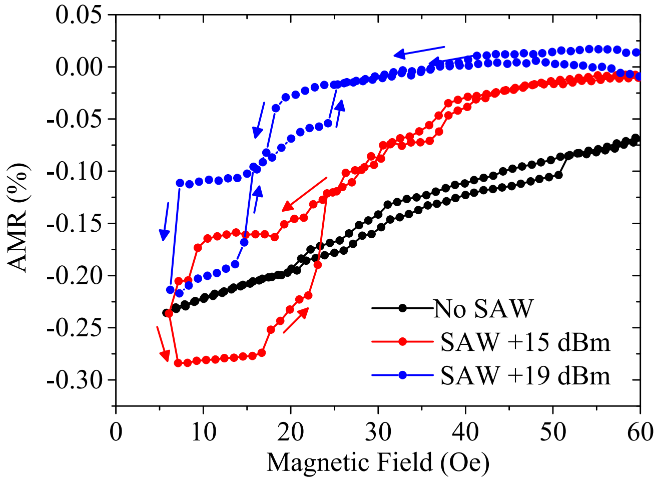

Figure S3.2. Minor loop from positive saturation to +6 Oe (not switching the direction of the external field) without SAW (black), with +15 dBm SAW (red) and with +19 dBm SAW (blue). The arrows indicate the direction of the loop.

S4. Direction of anisotropy.

Experimentally, we deposited the Ni nanostrip by sputtering with a magnetic field in the direction perpendicular to the nanostrip axis. The induced anisotropy at 90º, plus the shape anisotropy, results in an effective anisotropy at 55º.

Nevertheless, with our simulations we did check the effect of the direction of the in-plane anisotropy. The results of the simulations are displayed in Fig.S4. Only when the anisotropy is perpendicular to the nanowire axis, the hysteresis curves are strongly affected by the SAW and everything (domain configuration and hysteresis loops) match the experimental results.

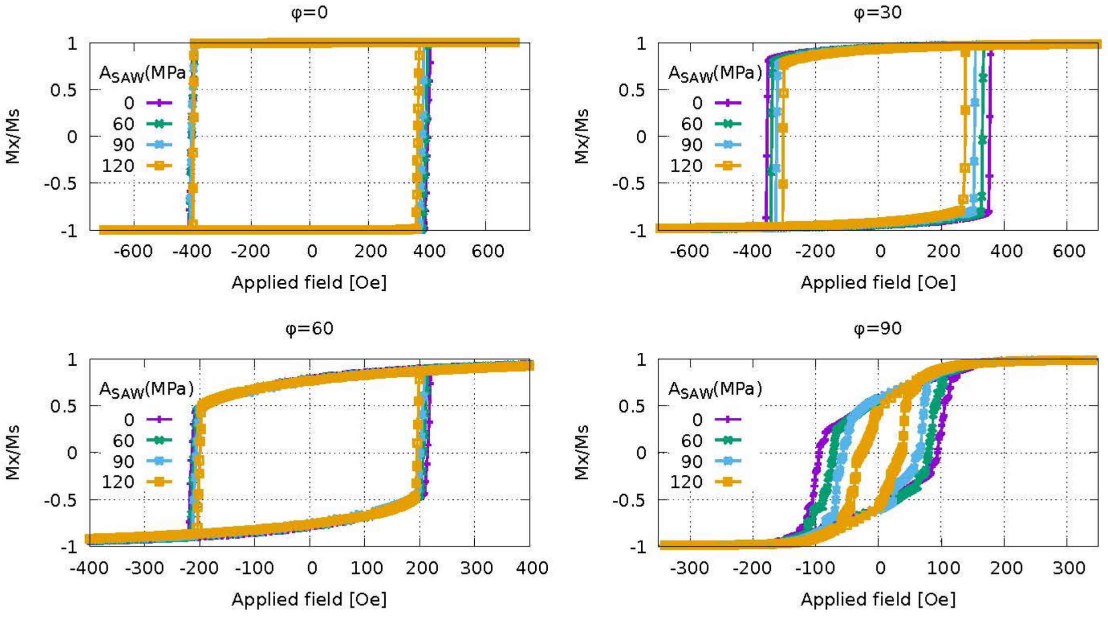

Figure S4. Hysteresis loops of the Ni nanostripe for different orientations of the in-plane anisotropy and different SAW amplitudes. From top to bottom and left to right: 0º, 30º, 60º and 90º.

S5. Integrating the SAW in the magnetization dynamics

The elastic energy per unit volume can be expressed as

$$\mathrm{U_{el}} = \frac{1}{2}\hat{\varepsilon}^{\mathrm{el}}\hat{\sigma} \quad (1)$$

where, $\hat{\varepsilon}^{\mathrm{el}}$ and $\hat{\sigma}$ are the elastic strain and stress tensors, respectively. They are related to each other by:

$$\hat{\sigma} = \hat{\mathrm{C}}\,\hat{\varepsilon}^{\mathrm{el}} \quad (2)$$

where $\hat{\mathrm{C}}$ is the fourth-rank elastic stiffness tensor. Due to magnetoelastic coupling, the total strain tensor has a magnetic component in addition to the elastic one, $\hat{\varepsilon} = \hat{\varepsilon}^{\mathrm{el}} + \hat{\varepsilon}^{\mathrm{m}}$, and therefore, Eq. (1) can be written as

$$\mathrm{U_{el}} = \frac{1}{2}(\hat{\varepsilon} - \hat{\varepsilon}^{m})\hat{C}(\hat{\varepsilon} - \hat{\varepsilon}^{m})$$

The magnetoelastic contribution to the effective field can be obtained from the elastic energy[1,2] as

$$\vec{H}_{me} = \frac{1}{\mu_0 M_s}\frac{\partial U_{el}}{\partial \vec{m}} = \frac{1}{\mu_0 M_s}\hat{\sigma}\frac{\partial \hat{\varepsilon}^m}{\partial \vec{m}}$$

which is the expression used in the main text (Eq. (2)).

S6. Amplitude and stress carried by the SAW.

The reflection parameter S_{11} of the IDT was determined as a function of the frequency using a vector network analyser, as shown in Figure S6. At the operation frequency f=1.19197 GHz, we recorded $|S_{11}(f)|$=0.02 (-33 dB). The electromechanical coupling factor was assumed 5% from Ref. S3 for a ScAlN device of similar dimensions.

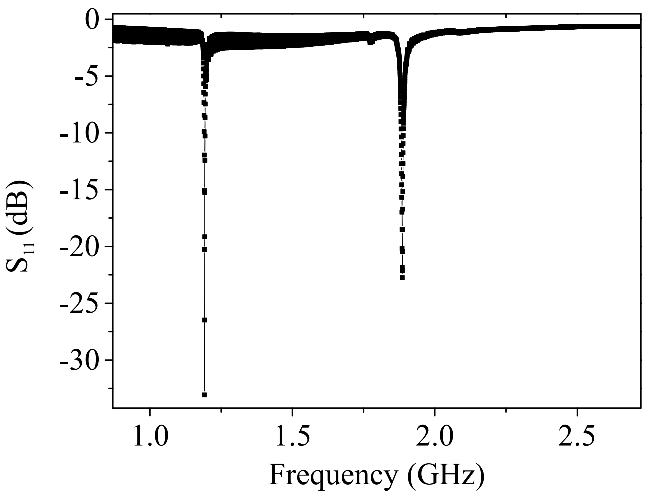

Figure S6. Reflection measurement of the IDT in the sample used for the study. We performed our study in the first resonance peak (Rayleigh).

In order to calculate the amplitude of the travelling SAW, we can use the textbook formula for the average power transmitted in 1D travelling waves,

$$\frac{1}{2}k^2 P = \frac{1}{2}A\rho v \xi_0^2 \omega^2$$

Where A is the area, ρ the density, v the velocity of sound, ξ_0 the amplitude of the wave and ω the angular frequency, P is the electric power and k^2 the electromechanical coupling coefficient[3] ($k^2 = 0.05$). The 1/2 on the left side of the formula accounts for the bidirectionality of the IDT. As we know the wavelength of the SAW (spacing of the IDT) and its frequency, we take $v=\lambda\cdot f=2.8\cdot 10^{-6}\cdot 1.2\cdot 10^{9}=$ 3360 m/s, which is very close to other values reported in the bibliography[3]. The density of ScAlN should be in the range of ρ=3500 kg/m^3 (see Table 1 in Ref.S4 of this Supplementary Information). Finally, the area is defined by the width of the IDT (100 μm) and the thickness of the ScAlN (2 μm), so $A=2\cdot 10^{-10}$ m^2. With these values, for a +19 dBm power (79.4 mW), we obtain an amplitude of the wave of ξ_0=0.17 nm.

To calculate the stress carried by the wave, we use Hook's law,

$$\sigma = Y \cdot \frac{\partial \xi}{\partial x}$$

The term $\partial\xi/\partial x$ is the strain, the derivative of the wave of amplitude, $\xi = \xi_0 \cdot \cos(2\pi x/\lambda - 2\pi f t)$. Therefore, the amplitude of the stress wave is simply,

$$\sigma_0 = Y \cdot \frac{2\pi\xi_0}{\lambda}$$

The Young modulus is assumed to be *Y*=220 GPa from Fig.6 of Ref. S5 (our samples have 43% Sc deposited at 500 W from a single target).

Power (dBm)	Power (mW)	Amplitude ξ_0 (Å)	Stress σ_0 (MPa)
0	1.00	0.19	9.5
5	3.16	0.34	17.0
10	10	0.61	30.2
15	31.6	1.09	53.7
19	79.4	1.72	85.1

Table S6.1. Conversion from dBm to amplitude of the displacement wave and amplitude of the stress wave

S7. Frequency dependence of the SAW.

We performed some simulations varying the frequency of the SAW and the results are presented in Fig. S7.1

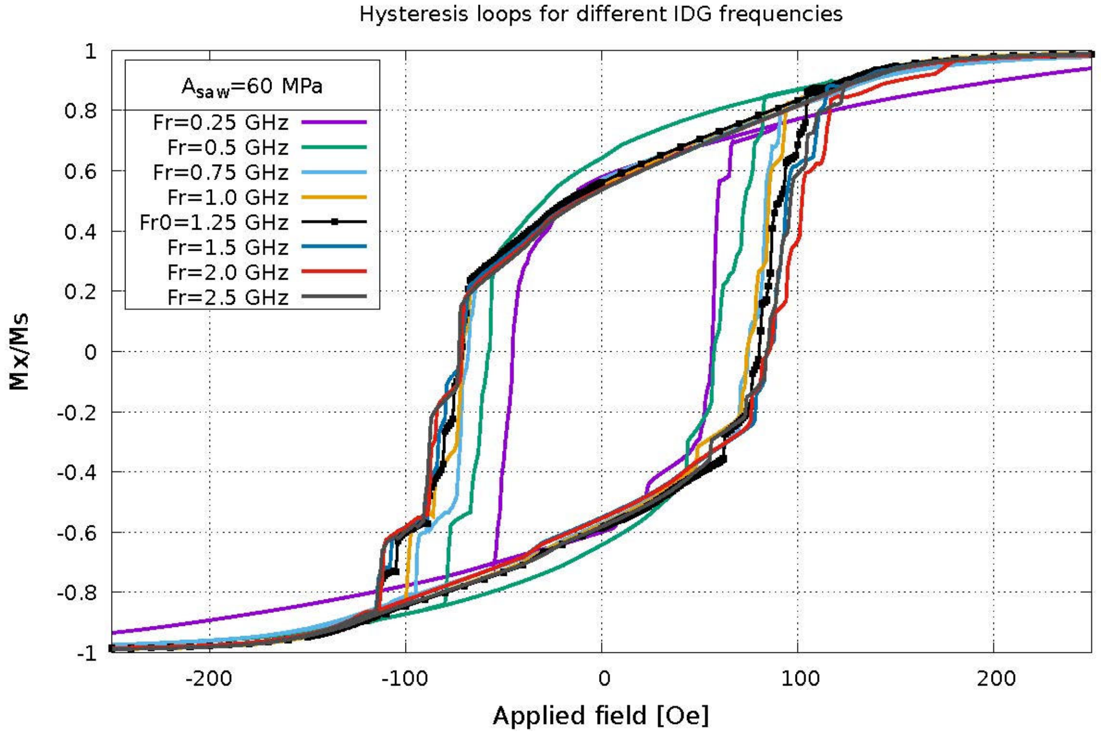

Figure S7.1. Hysteresis loops of the Ni nanostripe for different frequency values of the SAW (A = 60 MPa)

The effect of the SAW is qualitatively the same throughout the studied frequency range, decreasing the coercive field with the amplitude of the SAW for all frequencies. This reduction is more pronounced for lower frequencies than the one used in the experiment (f_{exp} = 1.2 GHz), as shown in Figure S7.1. A detailed study of why lower frequencies are more effective reducing the coercivity, or weather this would be a general trend or a particularity of our system, we believe is well beyond the scope of our work.

References

[1] C.-Y. Liang, S. M. Keller, A. E. Sepulveda, A. Bur, W.-Y. Sun, K. Wetzlar, and G. P. Carman. Modeling of magnetoelastic nanostructures with a fully coupled mechanical-micromagnetic model. Nanotechnology **25**, 435701 (2014).

[2] G. Consolo and G. Valenti. Analytical solution of the strain-controlled magnetic domain wall motion in bilayer piezoelectric/magnetostrictive nanostructures. J. Appl. Phys. **121**, 043903 (2017).

[3] G. Tang, T. Han, A. Teshigahara, T. Iwaki and K-y Hashimoto. Enhancement of effective electromechanical coupling factor by mass loading in layered SAW device structures. Joint Conference of the IEEE International Frequency Control Symposium & the European Frequency and Time Forum (2015). DOI: 10.1109/FCS.2015.7138870.

[4] Piezoelectric coefficients and spontaneous polarization of ScAlN. J. Phys.: Condens. Matter **27**, 245901 (2015)
